# Long extensions with varicosity-like structures in gonadotrope Lh cells facilitate clustering in medaka pituitary culture

**DOI:** 10.1371/journal.pone.0245462

**Published:** 2021-01-28

**Authors:** Heidi Kristine Grønlien, Romain Fontaine, Kjetil Hodne, Isabelle Tysseng, Eirill Ager-Wick, Finn-Arne Weltzien, Trude Marie Haug

**Affiliations:** 1 Faculty of Health and Welfare, Østfold University College, Halden, Norway; 2 Physiology Unit, Faculty of Veterinary Medicine, Norwegian University of Life Sciences, Oslo, Norway; 3 Department of Biosciences, Faculty of Natural Sciences, University of Oslo, Oslo, Norway; 4 Department of Oral Biology, Faculty of Dentistry, University of Oslo, Oslo, Norway; Universite de Rouen, FRANCE

## Abstract

Accumulating evidence indicates that some pituitary cell types are organized in complex networks in both mammals and fish. In this study, we have further investigated the previously described cellular extensions formed by the medaka (*Oryzias latipes*) luteinizing hormone gonadotropes (Lh cells). Extensions, several cell diameters long, with varicosity-like swellings, were common both *in vitro* and *in vivo*. Some extensions approached other Lh cells, while others were in close contact with blood vessels *in vivo*. Gnrh further stimulated extension development *in vitro*. Two types of extensions with different characteristics could be distinguished, and were classified as major or minor according to size, origin and cytoskeleton protein dependance. The varicosity-like swellings appeared on the major extensions and were dependent on both microtubules and actin filaments. Immunofluorescence revealed that Lhβ protein was mainly located in these swellings and at the extremity of the extensions. We then investigated whether these extensions contribute to network formation and clustering, by following their development in primary cultures. During the first two days in culture, the Lh cells grew long extensions that with time physically attached to other cells. Successively, tight cell clusters formed as cell somas that were connected via extensions migrated towards each other, while shortening their extensions. Laser photolysis of caged Ca^2+^ showed that Ca^2+^ signals originating in the soma propagated from the soma along the major extensions, being particularly visible in each swelling. Moreover, the Ca^2+^ signal could be transferred between densely clustered cells (sharing soma-soma border), but was not transferred via extensions to the connected cell. In summary, Lh gonadotropes in medaka display a complex cellular structure of hormone-containing extensions that are sensitive to Gnrh, and may be used for clustering and possibly hormone release, but do not seem to contribute to communication between cells themselves.

## 1. Introduction

The anterior pituitary is a complex endocrine gland that secretes multiple hormones to control different processes, like lactation, growth, reproduction, and homeostasis. Accumulating evidence indicates that several endocrine cell types are organized into complex three-dimensional homotypic and heterotypic networks in both mammals and teleost fish [1–7].

While the different pituitary cell types are distributed in a mosaic-like pattern in mammalian pituitaries [8], the different endocrine cell types are more clustered in teleost fish [9–11]. This is also the case for the gonadotropes which are located in the proximal *pars distalis* [9, 10, 12]. Gonadotropes play a major role in the control of reproduction by producing the gonadotropins—follicle-stimulating hormone (FSH) and luteinizing hormone (LH), which stimulate gametogenesis and steroidogenesis in the gonads [9]. In mammals and birds, FSH and LH are synthesized and secreted from the same cell [13], whereas in most teleost fish, they are produced by distinct cells [14, 15].

Gonadotropes have been described to form networks in several teleost species. In tilapia and zebrafish, Lh-producing gonadotropes (Lh cells) have been shown to be functionally coupled by gap junctions in soma-soma-contacts, forming a large, continuous network that permit intercellular communication through gap junctions [4, 16]. Uncoupling the gap junctions greatly reduces Lh release, indicating the importance of cellular networks for the total hormone release. The tilapia Fsh-producing gonadotropes (Fsh cells) appear to form a looser network via cellular extensions, rather than via gap junctions, and Fsh release was not affected by the gap junction uncoupler [4]. In medaka, both heterotypic and homotypic functional networks between closely adjoining Lh and Fsh cells have been demonstrated using uncaging and calcium imaging techniques [17].

Evidence is accumulating that a large variety of cells, not only neurons, form extensions that communicate with other cells by direct contact [18]. For teleosts, the extent and the putative roles of such extensions are barely described so far. Interestingly, previous studies have indicated that medaka Lh cells form extensions *in vitro* [19] and *in vivo* [20] and that these extensions seem to have a different appearance from those previously described in gonadotrope cells from other teleosts [4, 21, 22] and mammals [23–25]. This observation prompted us to investigate these extensions in more detail. A plethora of different names has been suggested for structures that may later be shown to be identical or similar. In this article, we have therefore chosen the neutral terms major and minor extensions.

Gonadotropes are under control of releasing and inhibiting factors from the brain—in mammals via the portal blood vessel system, in teleosts by axons extending to the pituitary target cells. The neuropeptide gonadotropin-releasing hormone (GnRH) is released in response to certain internal and external cues, and, in turn, stimulates pituitary gonadotropes. In mammals, it has been shown that GnRH triggers formation of gonadotrope extensions [23–25]. To our knowledge, similar effect of Gnrh has not been shown in any teleost before now. Despite the differences between mammalian and teleost pituitary architecture, teleost pituitary cells are important for comparative studies, as the neuroendocrine regulation and intracellular signaling pathways are similar [26].

Medaka (*Oryzias latipes*) is a small teleost fish with advanced molecular and genetic tools available to study its biology and physiology. In this study, we used a transgenic line where the green fluorescent protein (Gfp) is under the control of the medaka *lhb* promoter: tg(*lhb*:hrGfpII), and where it has previously been shown that Gfp is a good reporter not only for the presence of lhb mRNA, but also for the presence of Lhβ protein [27, 28]. We will thus refer to Gfp-positive cells as Lh cells in this paper. The endogenously produced Gfp in the tg(*lhb*-hrGfpII) medaka line provides a robust fluorescent signal from all parts of the cell, that remains during prolonged confocal imaging, and thus enables extensive and detailed mapping of anatomical structures within the tissue, as well as tiny, temporary filopodia-like structures in cell cultures.

Here we describe and define the nature of the extensions formed by Lh cells in medaka, both in intact pituitaries and dissociated primary cell cultures. In cell cultures, we have investigated dynamic properties not feasible to assess in intact tissue, like extension growth, formation of contact between cells, cell clustering, and how these extensions are affected by Gnrh.

## 2. Materials and methods

### 2.1 Animal model

Transgenic tg(*lhb*:hrGfpII) Japanese medaka (*Oryzias latipes*), in which expression of Gfp is controlled by the medaka *lhb* promotor [27], were used in all experiments. Fish were kept in recirculating water systems with water temperature between 25 and 28°C, constant salinity (800 μS) and pH (7.6), and light-dark cycle of L14:D10. Fish were fed three times daily on a combination of dry feed (Scientific Fish Food, Special Diets Service, Essex, UK) and newly hatched brine shrimp, *Artemia sp*. (Argent, Redmond, WA, USA). Fish used in the experiments were one-year old females, except for imaging of intact pituitaries where both sexes where studied. Handling and use of fish were in accordance with guidelines of the Animal Welfare Committee of the University of Oslo.

### 2.2 Preparation of whole pituitary glands

To investigate whether Lh cells possess extensions *in vivo*, 21 pituitaries (from 14 female and 7 male fish) were removed rapidly from the skulls of anesthetized (0.2–0.3 mg/ml benzocaine), decapitated medaka and embedded in a drop of 2% low-melting point agarose (Sigma-Aldrich, St. Lois, MO, USA) in the grooves (5 mm in diameter) of cavity microscope slides. The agarose was dissolved in artificial extracellular solution (ECS) containing 134 mM NaCl, 2.9 mM KCl, 2.1 mM CaCl_2_, 1.2 mM MgCl_2_, 0.1% bovine serum albumin (BSA), 10 mM HEPES, and 1.8 mM glucose. pH was adjusted to 7.75 with NaOH, and the osmolarity adjusted to 280 mOsm using mannitol. Once the agarose had solidified, ECS at 26°C was added on top to keep the agarose moist before confocal imaging. All images were taken within 30 min after dissection, to ensure that the tissue was still alive and of high quality.

### 2.3 Labeling of pituitary blood vessels

To visualize blood vessels, lipophilic carbocyanine dye (DiI) (Invitrogen, Carlsbad, CA, USA), diluted in 4% paraformaldehyde (PFA) in phosphate buffered saline (PBS), was introduced into anesthetized fish by cardiac perfusion, as previously described in detail [29]. Brain and pituitary were subsequently dissected and fixed overnight at 4°C in 4% PFA. Tissues were then rinsed with PBS, embedded in 3% agarose, and sectioned with a vibratome (Leica, Wetzlar, Germany). Sections were stained with 4’6-diamidino-2-phenylindole dihydrochloride (DAPI; 1/1000, Sigma-Aldrich) for 20 min and mounted using vectashield (Vector Laboratories, Peterborough, UK) between glass and coverslip.

### 2.4 Preparation of primary pituitary cell cultures

Cell cultures were made as previously described [19, 30]. In brief, pituitaries were dissected from anesthetized fish and enzymatically digested with trypsin (2 mg/mL, Sigma-Aldrich) for 30 min at 26°C, followed by incubation with trypsin inhibitor (1 mg/mL, Sigma-Aldrich) and Dnase I Type IV (2 μg/mL, Sigma-Aldrich) for 20 min at 26°C with gentle shaking.

Pituitary cells were then mechanically dissociated using a glass pipette, centrifuged at 100 *g* and resuspended in growth medium (L-15, Life Technologies) supplemented with 10 mM NaHCO_3_ (in order to stabilize pH at 7.75), 1.8 mM glucose, penicillin/streptomycin (5 000 U per 100 mL medium, Lonza, Verviers, Belgium) and adjusted to 280–290 mOsm with mannitol. Dissociated cells were plated on poly-L-lysine pre-coated dishes fitted with a central glass bottom (MatTek Corporation, Ashland, MA, USA). Each dish contained about 50 000–100 000 cells, limited to the glass region of the dish. The cells were incubated at 26°C and 1% CO_2_, and used in experiments for up to three days after seeding, but to study the initial movements of Lh cells and development of their extensions, time-lapse confocal imaging was conducted immediately after seeding.

### 2.5 Immunofluorescence

Immunofluorescence (IF) in wild type medaka was performed on brain-pituitary slices and dissociated pituitary cells two days after plating. For IF on slices, brain-pituitary complexes were collected and fixed in 4% PFA overnight at 4°C before sectioning was made with a vibratome (Leica). IF on dissociated cells was performed in two different dishes, fixed in 4% PFA for 10 min at RT. After fixation, tissue slices and dissociated cells were rinsed in PBS-Tween (PBST) several times, and incubated in blocking buffer [10% normal goat serum (Sigma-Aldrich) in PBST] for 1 h. Half of the sections and one dish was then incubated overnight at 4°C with a rabbit anti-medaka Lhβ-antibody (1/1000 in blocking buffer) previously used and validated on slices [31] and dissociated cells [20], while the other half of the sections and the other dish were incubated in blocking buffer without the antibody. Tissues and cells were then washed in PBST three times, and incubated for 1 h with secondary antibody, Goat anti Rabbit conjugated to Alexa-fluor 488 (1/500, Invitrogen), at RT before being washed again in PBST several times before imaging.

### 2.6 Exposure of primary pituitary cell culture to cytoskeleton inhibitors

To determine the cytoskeleton architecture of extensions, cells were exposed to either an inhibitor of actin filaments, cytochalasin B (Sigma-Aldrich), or to a tubulin-polymerization inhibitor, nocodazole (Sigma-Aldrich). Two hours after plating the cells, cytochalasin B or nocodazole [1:5000 dilution from a 10^−2^ M stock in DMSO] was added, giving a final concentration of 10^−7^ M. The cells were incubated at RT with cytochalasin B and nocodazole for 2 h followed by live imaging for 4 h. The experiments were performed from 3 independent cultures and the controls were cells exposed to growth medium containing the same concentration of DMSO at RT for similar time interval as the drug-exposed cells.

### 2.7 Uncaging of Ca^2+^ in primary pituitary cell cultures

One to two days after plating, cells were incubated for 1 h at 27°C with 0.01% pluronic, 5 μM o-Nitrophenyl EGTA, AM (NP-EGTA-AM, Ca^2+^ caging compound, Thermo Fisher Scientific) and 5 μM Cal-590-AM (Ca^2+^-sensitive dye, AAT Bioques, Sunnyvale, CA, USA) in ECS without BSA, followed by 20 min de-esterification of NP-EGTA and Cal590 in ECS containing 0.1% BSA. Cal590 was excited at 580 nm. NP-EGTA was uncaged using pulses of 50–250 ms delivered with a 405 nm laser pre-set at 7 mW (Laser Applied Stimulation and Uncaging system, Scientifica), passing through an 80/20 beam splitter, and targeting distinct regions of the cells using two galvanometer scan mirrors controlled by a Scientifica software developed in LabVIEW (National instruments, Austin, TX, USA). The experiments were performed from 4 independent cultures and the control experiments ensured that when targeting the laser to one cell soma, the neighbor cells were not affected (S1 Fig).

### 2.8 Gnrh treatment of primary pituitary cell culture

Gnrh1 (Bachem, Budendorf, Switzerland) was added to the growth medium one day after plating the cells, to give a final concentration of 10^−7^ M. This is a concentration used previously in studies on both teleosts [19, 32] and mammals [24, 25] and shown to elicit robust responses. The cells were exposed to Gnrh1 and followed in time-lapses for up to 20 h. The experiments were performed from 8 independent cultures and the control cells were incubated in growth medium for similar time interval as the Gnrh1-exposed cells.

### 2.9 Imaging

Whole pituitaries, and most of pituitary sections and primary pituitary cell cultures were imaged using an Olympus FluoView1000 inverted IX81 confocal laser-scanning microscope (Olympus, Tokyo, Japan) with water immersion 4X, 20X, and 40X, or oil 60X (1.10 N.A. Plan APO) objectives as appropriate, or Zeiss LSM710 confocal microscope (Carl Zeiss AG, Oberkochen, Germany) with 63X objective (1.3 N.A. LCI Plan-Neofluar). Optical sections were made every 0.2–0.5 μm along the z-axis and images were obtained through projections of the z-stack. Time series was obtained by taking a picture every 0.5–10 min for 10–48 hours after dissociation, depending on the experiment. The cell cultures were also visualized using Olympus IX72 inverted microscope with water immersion 20X objective. Emission was recorded using Hamamatsu ORCA ER camera (Hamamatsu Photonics, Hamamatsu, Japan).

The cytosolic calcium was visualized using an infrared Dodt Gradient Contrast (DGC) system coupled to an upright Olympus fluorescence microscope with 40X water immersion objective (0.8 N.A. Slicescope, Scientifica, Uckfield, UK). For excitation of Cal590, a diode light source was used (pE-4000 CoolLED, Andover, UK) at 550 nm, and the emission was collected after passing a 630/75 bandpass filter (Chroma Technology Corp, Vermont, USA). For Ca^2+^ imaging, an exposure time of between 50 and 80 ms was used and images were sampled at 0.5 Hz. The cells were imaged using a sCMOS camera (optiMOS, QImaging, Surrey, BC, Canada) and both the light source and camera were controlled by μManger software, version 1.4 [33]. Image analysis was performed in Fiji [34]. For the uncaging calcium experiments, images containing laser reflection were removed from the final imaging profile post recording and the relative fluorescence intensity was calculated after background subtraction as changes in fluorescence (ΔF) divided by average intensity of the first 15 frames (F).

### 2.10 Measurements, image analysis and statistical analysis

Images were processed using the open-source software from ImageJ (versions 1.37v and 2.0.0, National Institute of Health, Bethesda, MD, USA) and Imaris 9.1 (Bitplane, Oxford Instruments Company, Abingdon, UK). All figures were made in Adobe Photoshop, Illustrator, and Indesign CC2018 (Adobe systems Inc., San Jose, CA, USA).

The lengths of extensions were measured with the ImageJ plugin NeuronJ [35] as shown in Fig 2C. For extensions running between two cells, the length of each extension was determined as half the total length. The smallest diameter was used for analysis.

All data from primary cell cultures are from at least three independent cultures. The data are presented as mean ± standard deviation (SD) or median with interquartile range (IQR). Preliminary analyses were performed to ensure no violation of the assumptions set by each statistical test. Independent sample t-tests were performed for comparisons of the normally distributed data of length, diameter and number of extensions, and to account for non-normality distribution, Mann-Whitney U-tests were performed for comparison of time. To compare the means of length and diameter of the extensions on cells treated with cytoskeleton inhibitors, one-way analysis of variance (ANOVA) followed by Tukey post hoc test was used. The relationship between minor extensions on major extensions was investigated using Pearson’s product moment correlation coefficient (r). *p* values below 0.05 were defined as statistically significant for all tests. All analyses were performed using SPSS 23 (IBM, Armonk, NY, USA). Boxplots were generated by the application BoxPlotR [36].

## 3. Results

### 3.1 Lh cell extensions in intact pituitaries

**Fig 1:**
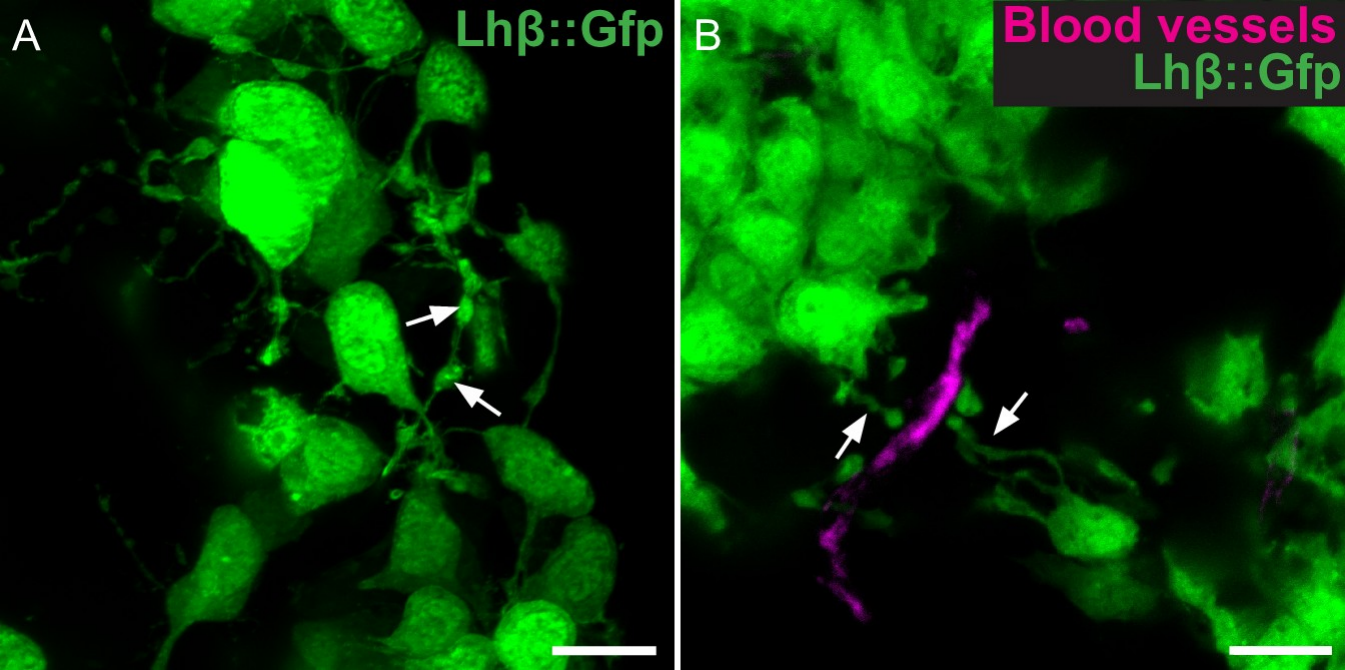
Lh cell extensions in whole pituitary. (A) Projection of 10 μm confocal z-stack of Gfp (Lh) cells in freshly dissected, intact pituitary. Arrows show swellings. (B) Projections of 10 μm confocal z-stack of Gfp (Lh) cells in a fixed pituitary section with DiI cardiac perfusion preceding dissection (DiI labels endothelial cells). Arrows show swellings nearby a blood vessel. Scale bars represent 10 μm.

We first investigated whether the long extensions previously observed in cultures could also be observed *in vivo*. Confocal images from freshly dissected, intact pituitaries revealed that Lh cells form numerous long extensions (Fig 1A). Interestingly, the extensions displayed multiple distinct areas of enlargement, hereafter referred to as swellings (arrows). Similar structures were observed in intact pituitaries from both males and females. Images from pituitary sections revealed that many of the extensions are reaching towards other Lh cells. Some others ended in close proximity to blood vessels, with their terminal part wider where it reached the blood vessel (Fig 1B).

### 3.2 Lh cells display two types of extensions in primary culture

**Fig 2:**
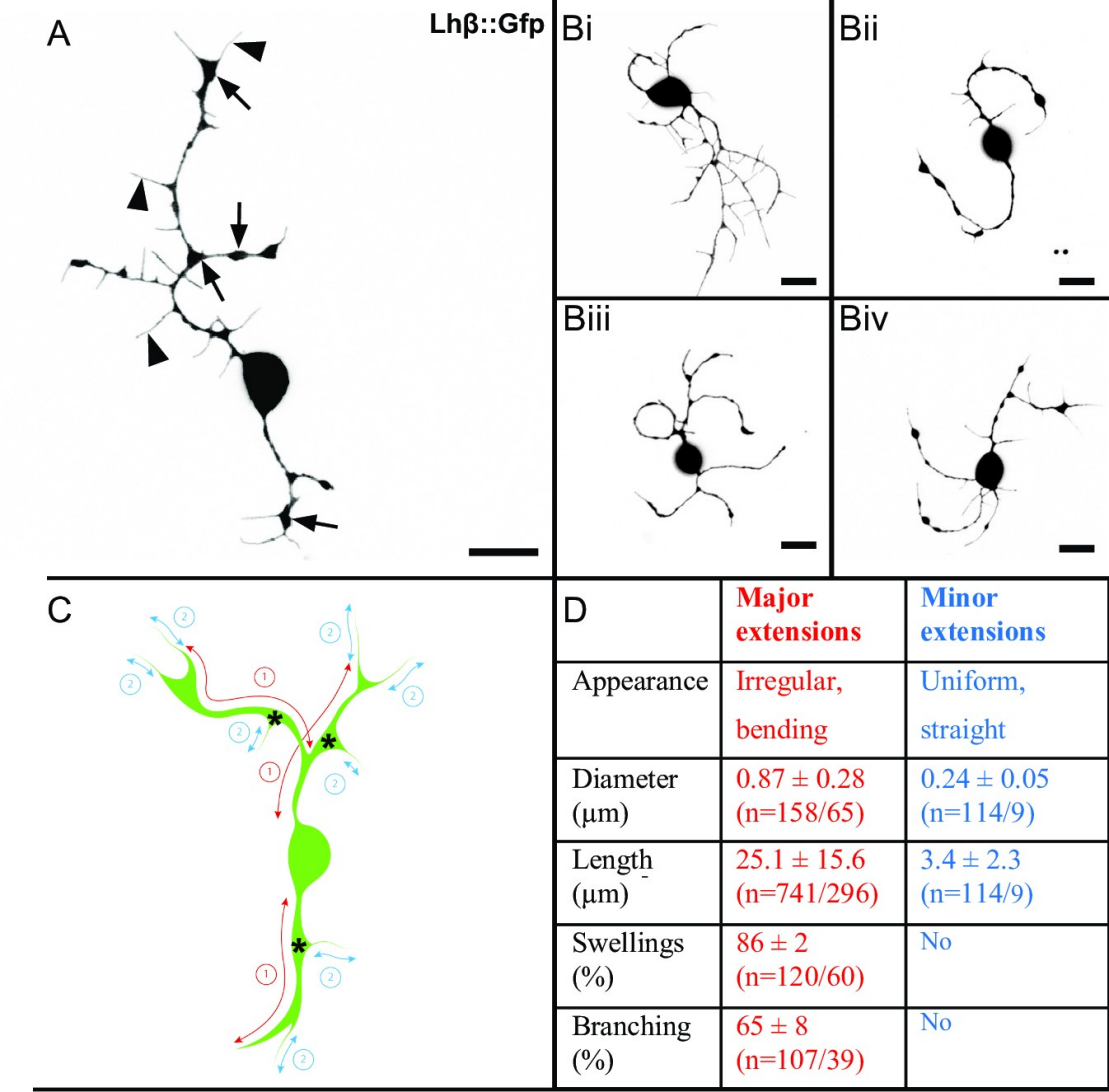
Lh cells with major and minor extensions in primary cell culture. (A) Projection of a confocal plane of an Lh cell from 48 h old dissociated primary pituitary cell culture. Arrows show swellings on major extensions and arrowheads show minor extensions. (Bi-iv) Set of planar confocal projections of Lh cells from primary cell cultures. Images present the heterogeneity of the Lh cells. Scale bars represent 10 μm. (C) Scheme of an Lh cell describing mapping of the extensions. Red arrows (1) indicate major extensions, and blue arrows (2) indicate minor extensions. Each extension was classified as either, depending on size and general appearance. (D) Characteristics of major and minor extensions of Lh cells in primary cell cultures. The analyses are from cells 48 h after seeding. All values are from three independent cultures and presented as mean ± SD (n = extensions/cells).

Because it is difficult to investigate details of the extensions in the whole pituitary where they are often hidden by the strong fluorescence of neighboring cell bodies, we further characterized their structure and development in primary pituitary cell cultures. Although the cells lose their extensions during dissociation, Lh cells start forming new extensions after approximately 1 h (median 66 min, IQR 128 min, n = 45). After 48 h in culture, extensions were observed from 98 ± 2% of the Lh cells (n = 311) and displayed a high morphological heterogeneity (Fig 2A and 2B). Extensions were also observed from 20 ± 10% of the non-Lh cells (n = 517), but these were not investigated further.

According to appearance, two distinct types of extensions were identified: hereafter named major and minor extensions (numbered 1 and 2 in Fig 2C, respectively). Fig 2A shows a representative cell with major and minor extensions and the table in Fig 2D presents the descriptive values from the two types of extension. The major extensions were heterogenic and varied in length with the longest up to 100 μm. 65% of the major extensions that originated from the soma branched into two equal extensions. Swellings were visible in 85% of the major extensions, and were regularly spaced, with mean distance 10.9 ± 5.8 μm (n = 60 cells/103 extensions). The mean cross-sectional area of a swelling constituted 6.8 ± 3.8% of the area of the soma (n = 60 cells/103 extensions).

The minor extensions were relatively straight and uniform in thickness and without swellings and branching (Fig 2A). They were significantly thinner (*p*<0.001) compared to major extensions, and none of the minor extensions exceeded 13 μm in length. Minor extensions were growing from all regions of the major extensions, including the tip and swellings. For each major extension, 6.9 ± 3.4 minor extensions (n = 16, in 9 cells) were observed. The number of minor extensions correlated with the length of their major extension (r = 0.597, n = 16, *p* = 0.015).

### 3.3 Lh cell extensions contain Lhβ protein

**Fig 3:**
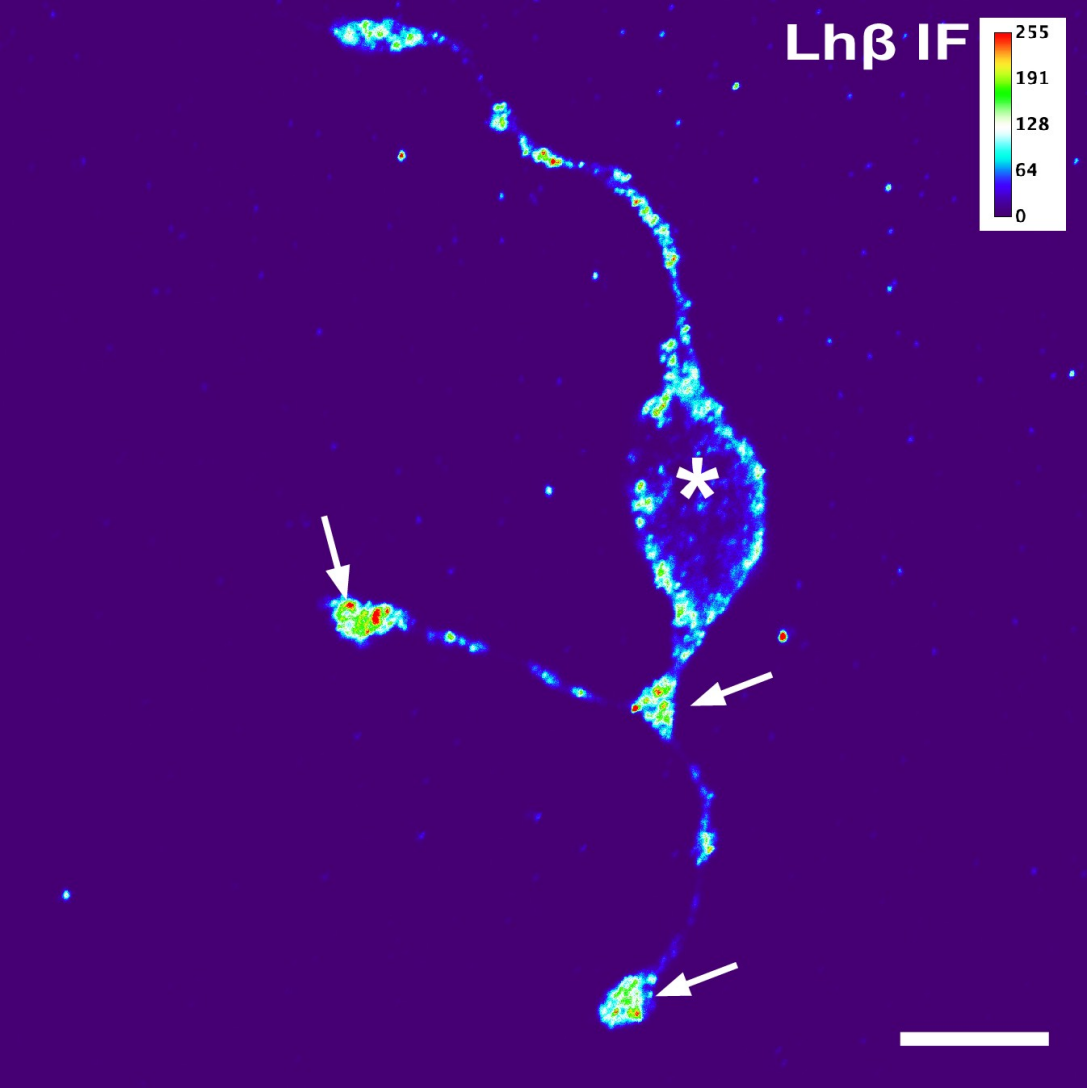
Lhβ proteins in Lh cells in primary cell culture from wild type medaka. Heatmap from a planar confocal projection of a cell labelled for Lhβ by immunofluorescence. Arrows show the swellings and the extremities of the extensions with high content of Lhβ protein, and asterisk shows the cell body. Scale bar represents 10 μm.

Although the previous figures clearly show that the extensions and swellings are filled with Gfp, this does not imply that the Lh hormone is located here, as the Gfp protein is not linked to the Lhβ protein. Therefore, to visualize the location and distribution of the hormone itself, immunofluorescent labeling of the Lhβ protein was performed in primary culture from wild type medaka. First, when incubating the cells with only either the primary or secondary antibody, no labeling was observed, confirming the specificity of the secondary antibody. Second, high fluorescence was detected in the major extensions, with the swellings and the extremities of the extensions being more strongly labelled than the cell soma (Fig 3), suggesting an accumulation of Lhβ protein in these cellular regions. The lower labelling intensity of the extensions between swellings may simply be due to the smaller volume, and thus a lower amount of Lh-containing vesicles. Similar staining of Lh cells with extensions and swellings was observed also in pituitary tissue slices (S2 Fig).

### 3.4 Cytoskeleton architecture of Lh cell extensions

**Fig 4:**
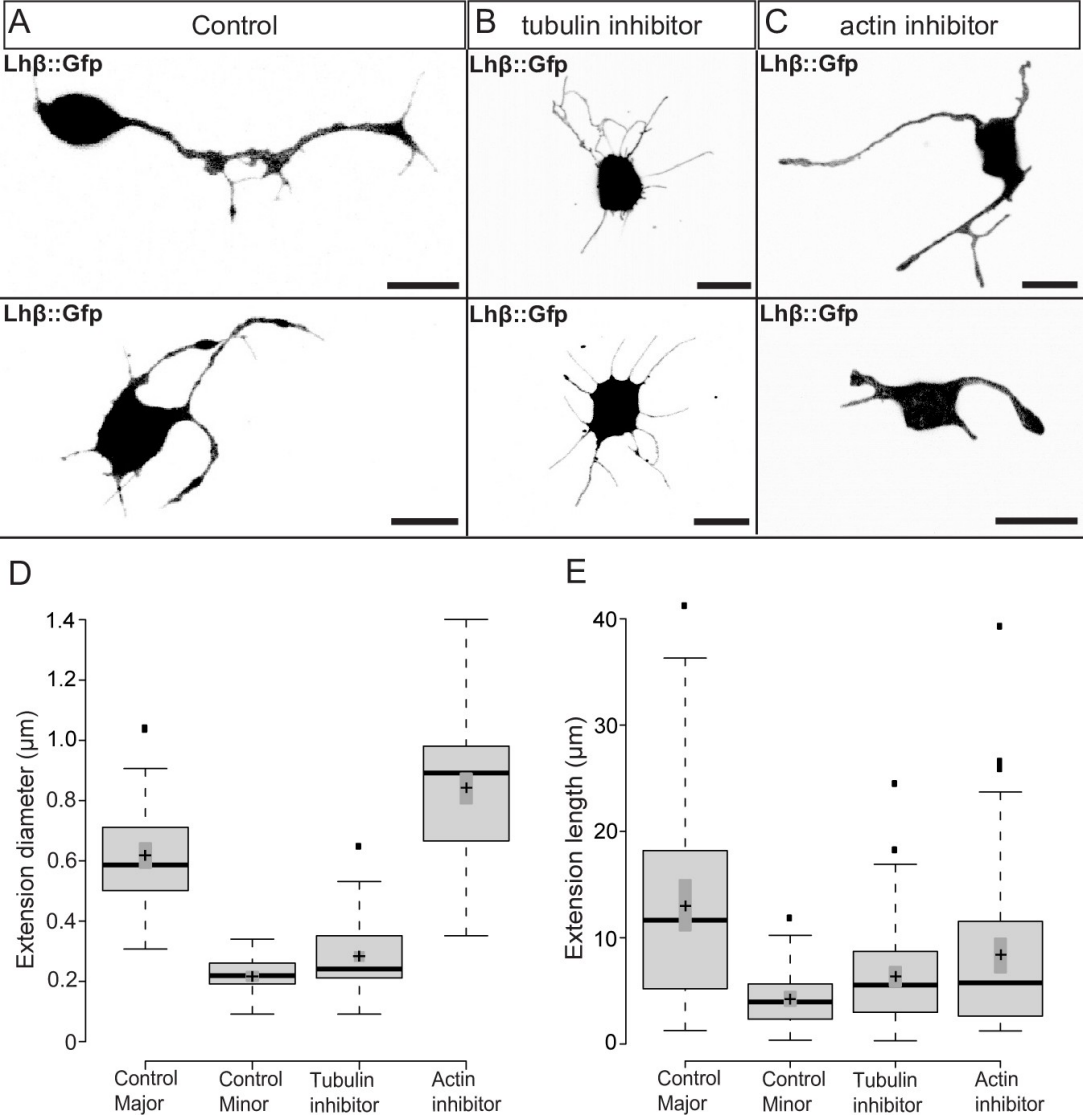
Lh cell extensions in culture treated with cytoskeleton inhibitors. Planar confocal projections of (Ai-ii) Lh cells with DMSO only (control), (Bi-ii) Lh cells treated with 10^−7^ M of the actin filament inhibitor cytochalasin B, and of (Ci-ii) Lh cells treated with 10^−7^ M of the microtubules inhibitor nocodazole, for 4 h (images taken 6 h after seeding). Scale bars represent 10 μm. (D-E) Boxplot of extension diameter and length 6 h after seeding. Center lines show the medians; box limits indicate the 25th and 75th percentiles as determined by R software; whiskers extend 1.5 times the interquartile range from the 25th and 75th percentiles, outliers are represented by dots; crosses represent sample means; bars indicate 95% confidence intervals of the means. n = 66, 69, 100, 84 extensions. *p* values were calculated using ANOVA with Tukey post hoc test. There was significance at .05 level between all groups except between the means of length of minor extensions (in control) and the extensions when treated with microtubule inhibitor. At .001 level, the mean difference both in length and diameter were significant between major extensions (in control) and the extensions treated with inhibitors for actin and for microtubules, respectively. There were significant differences at a .001 level between the means of both length and diameter for minor extensions (in control) and the extensions treated with actin inhibitor.

To investigate the cytoskeleton composition of Lh cell extensions, cells were treated either with DMSO only (control), nocodazole (a tubulin polymerization inhibitor), or cytochalasin B (an inhibitor of actin filaments). Control cells with DMSO displayed major and minor extensions similar to non-treated cells (Fig 4A). Lh cells incubated with cytochalasin B, produced extensions with diameter similar to major extensions (Fig 4D), without the distinctive swellings, and in 22% of the cytochalasin B treated cells the extensions branched (Fig 4B and S1 Table). Only 6% of the extensions were straight. In addition, the somas generally displayed an irregular shape. Following incubation with nocodazole, the Lh cells produced extensions without the distinct swellings, only 29% were branched, and the extensions were uniform in thickness with a diameter similar to minor extensions (Fig 4C, 4D and S1 Table).

### 3.5 Contacts between Lh cells formed by their extensions

**Fig 5:**
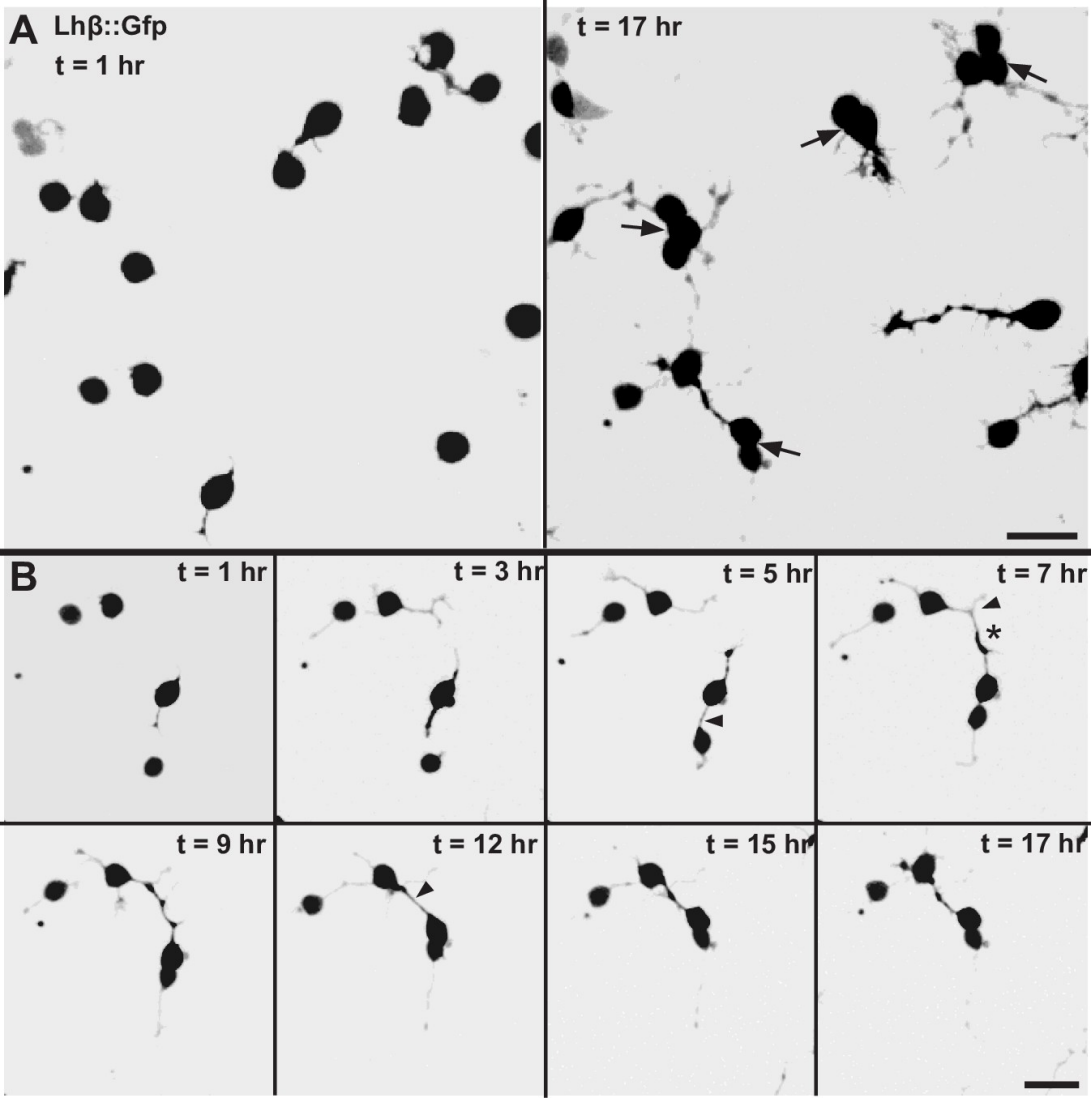
Development of extensions and contact with surrounding Lh cells in primary cell culture. (A) Planar confocal projections of Lh cells immediately after seeding (0 h) and 17 h after seeding. Arrows show Lh cells clustered in groups. (B) Time-lapse projections of four Lh cells. Time zero is immediately after seeding. Arrowheads show extensions making contact followed by formation of a bridge. Asterisk marks a non-Lh cell. See also S1 Video. Scale bars represent 10 μm.

We followed the cells in time-lapse recordings from directly after seeding for approximately 17 h (Fig 5 and S1 Video). The growth of the extensions, both the minor and major, during the first day in primary culture was dynamic; the individual extensions alternately extended, retracted or were at standstill. When reaching another Lh extension or soma, seemingly physical interactions were often made. In 44% of the analyzed interactions between two Lh cells (n = 16), contacts were made by an extension from one Lh cell connecting with an extension from the other Lh cell (Fig 5B time = 7 h, and S1 Video). The mean distance between the somas of the Lh cells at the point of physical contact was 27.2 ± 9.8 μm (n = 7), and the median time from when the extensions were 10 μm from each other until contact was established was 6.0 min (IQR = 3.0 min, n = 7). Contact could also be made directly between an extension and the soma of another Lh cell (Fig 5B time = 5 h and S1 Video). Here, the mean distance between the somas of the two Lh cells was 13.7 ± 4.7 μm (n = 9). Thus, the distance was significantly shorter than when two extensions from different Lh cells connected (*p*<0.001). The median time from when the extension was 10 μm from the other cell’s soma to connection was made was 12.0 min (IQR = 12.0, n = 9), which is significantly longer than when two extensions the same distance apart connect (*p* = 0.04).

**Fig 6:**
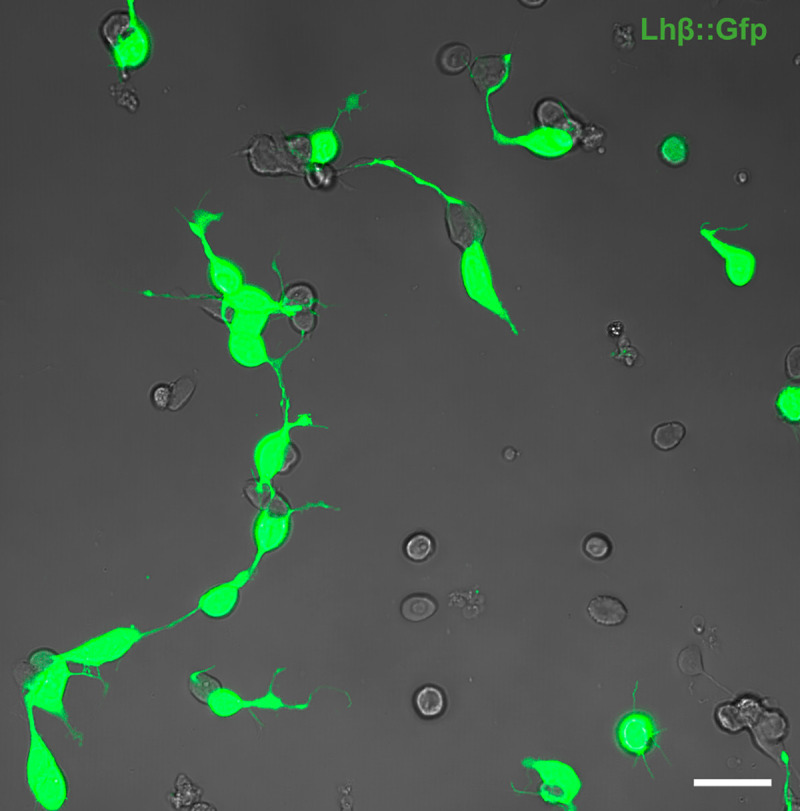
Lh cells make contacts through their extensions. f Planar confocal projection merged with the transmitted-light image showing bothLh cells (green) and non-Lh cells together in a 24 h old primary culture. Scale bar represents 10 μm.

We also observed contacts via extensions between Lh cells and non-Lh cells, as well as between non-Lh cells only (Fig 6 and S2 Video). In the time-lapse images analyzed, some Lh cell extensions seemed to contact non-Lh cells, but the extension was immediately withdrawn. In addition, an Lh cell extension could orbit the soma of a non-Lh cell and contact another Lh cell further away, as seen in Fig 5B. For an overview of the number of observed contacts made between Lh and non-Lh cells during the first 15 h in culture, see S3 Fig. Based on fluorescence microscopy images from three-day cultures (n = 1530 cells, 750 Lh cells), we found that 50 ± 7% of the cells were Lh cells (n = 3 cultures). Of all the 285 contacts made by extensions, we estimate that only 15 ± 2% were between an Lh cell and non-Lh cell, and 7 ± 5% of the contacts were between non-Lh cells. The rest, 77 ± 3%, were connections between two Lh cells. Due to the lack of Gfp staining in non-Lh cells, there is a larger degree of uncertainty as to the nature and solidity of these contacts, and whether they are indeed connected.

### 3.6 Clustering of Lh cells following connection by extensions

Both ways of contact between two Lh cells, either extension-extension or extension-soma, resulted in formation of a continuous structure without swellings that connected the two somas, hereafter referred to as a bridge (indicated by arrowheads in Fig 5B). The bridges typically straightened after contact, with median time between contact and straightening of the bridge being 12.0 min (IQR = 25.5 min, n = 16). The bridges were dynamic in nature; following the cells’ movements without breaking (Fig 5B time = 12 h and S1 Video), and subsequently bringing the Lh cells’ somas closer together. During the 17 h of the time lapse recordings, 50% of the bridges in a cell pair (n = 16) shortened all the way until the two somas met, thus initiating clustering. We never observed that two cells clustered without making any extensions first. The mean diameter of each bridge in a cell pair was 1.05 ± 0.27 μm (n = 16), which is significantly thicker than the diameter of a major extension (*p*<0.001).

To get an overall picture of the dynamics of clustering including all pituitary cell types, we followed the clustering process during the first 15 hours after seeding (S4 Fig). Many clusters were present already at time = 0 (due to incomplete dissociation), but of the new clusters that actively formed during the time-lapse, most were homotypic Lh cell clusters, however heterotypic clustering was also observed (see S2 Video). The number of single Lh cells diminished as the number of Lh cells in homotypic clusters increased. It is common for cells in culture to move and cluster, and we observed that, based on fluorescence microscopy images, 55 ± 2% of the cells (n = 1530 cells, 750 Lh cells) were clustered after three days in culture. The clusters were of three types; homotypic with Lh cells (n = 110, 261 Lh cells), heterotypic with Lh cells and non-Lh cells (n = 111, 190 Lh cells and 149 non-Lh cells), and clusters without Lh cells (n = 107, 294 non-Lh cells). Due to high density of cells in cluster, 11 clusters were discarded in the analysis. It has to be noted that in our three-day primary cell cultures, we cannot assume that the cells were dissociated completely and evenly dispersed by seeding, thus, quantification of the clustering process based on still images from day three is not performed.

### 3.7 Ca^2+^ signals transfer between cells in cluster, but not via extensions

**Fig 7:**
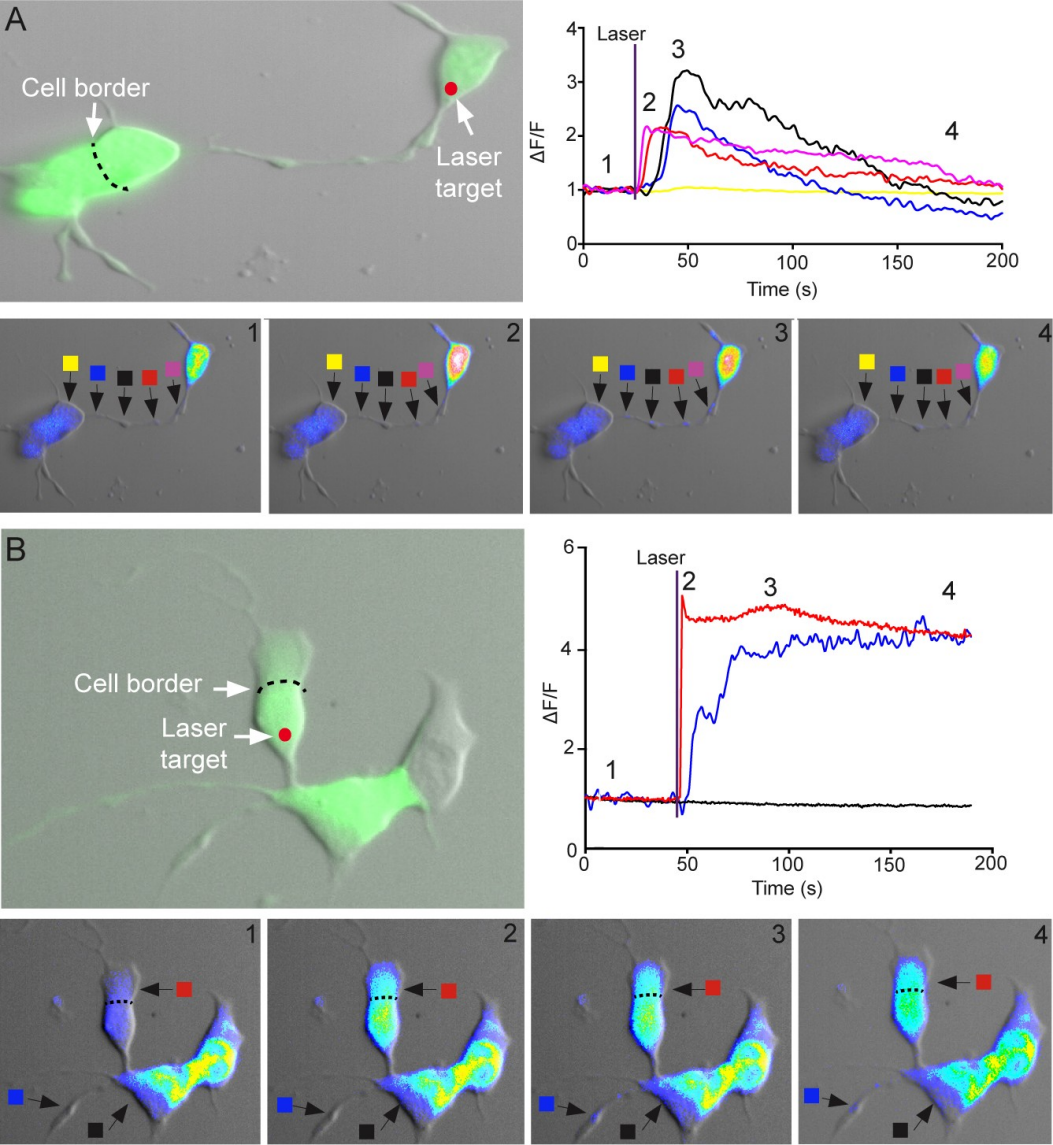
Ca^2+^ signals in Lh cell extensions and between clustered Lh cells in primary cell culture. Flash photolysis of caged Ca^2+^ in cultured Lh cells recorded by Ca^2+^ imaging. (A) Upper left panel shows two Lh cells connected by an extension. The area of Ca^2+^ uncaging is marked with an arrow; colored boxes indicate the sites of intracellular Ca^2+^ measurement shown in the upper right panel. Changes in fluorescence (ΔF) divided by the average intensity of the first 15 frames (F) as a function of time. The pictures in the lower panels show imaging of intracellular Ca^2+^ concentration at the four time points indicated in the upper right graph. Note that the Ca^2+^ signal propagates along the extension, but does not transfer to the connected cell. See also S3 Video. (B) Upper left panel shows two Lh cells connected by a bridge. The upper cell is clustered with another Lh cell, soma to soma. The area of Ca^2+^ uncaging is marked with an arrow; colored boxes indicate the sites of intracellular Ca^2+^ measurement shown in the upper right panel. Changes in fluorescence (ΔF) divided by the average intensity of the first 15 frames (F) as a function of time. The pictures in the lower panels show imaging of intracellular Ca^2+^ concentration at the four time points indicated in the upper right graph. Note that the Ca^2+^ signal transfers across the soma-soma border, but not across the bridge. See also S4 Video.

To examine if Ca^2+^ signals can propagate through the extensions, and subsequently transfer to the connected cell, Ca^2+^ was uncaged in the soma of one Lh cell. Two cells were assumed to be connected if we could not observe any gap in the combined structure, and the extension between the two cells clearly started/ended at the somas. Monitoring the cytosolic Ca^2+^ concentration showed clearly that the Ca^2+^ signal propagated along the major extensions from the soma to the terminal of the extension (Fig 7A and S3 Video). In particular, Ca^2+^ was transiently, but significantly increased in each swelling along the extension, with a time delay of 1–5 s depending on the distance from soma. However, we did not observe any increase of intracellular Ca^2+^ in the second cell in the connected pairs, neither via regular extensions (n = 20) nor via straight bridges (n = 13) (Fig 7B). Occasionally (n = 3), we observed an elevation in intracellular Ca^2+^ concentration in extensions that we were unable to assign to neither the stimulated cell nor its neighbor (Fig 7B, blue square and S4 Video). When uncaging was performed in the extensions, we did not observe any backpropagation of the Ca^2+^ signal to the soma (data not shown). Furthermore, cells in close proximity (less than one cell diameter) did not respond when uncaging the surrounding cells. In contrast, when two or more cells were clustered, uncaging of Ca^2+^ in the soma of one cell induced a significant elevation of cytosolic Ca^2+^ in the soma of one or more of its direct neighbor cells in 71% of the experiments (n = 21) (Fig 7B, red square and S4 Video). The elevation of Ca^2+^ in the neighbor cell occurred within 500 ms after uncaging Ca^2+^, and the peak response normally occurred 1–2 s after uncaging. Taken together, these results may suggest that the mechanism(s) reinforcing the Ca^2+^ signal from soma to soma and throughout the extension are solely intracellular and located in the soma of the cell itself.

### 3.8 Morphological effects in Lh cells following Gnrh1 exposure

**Fig 8:**
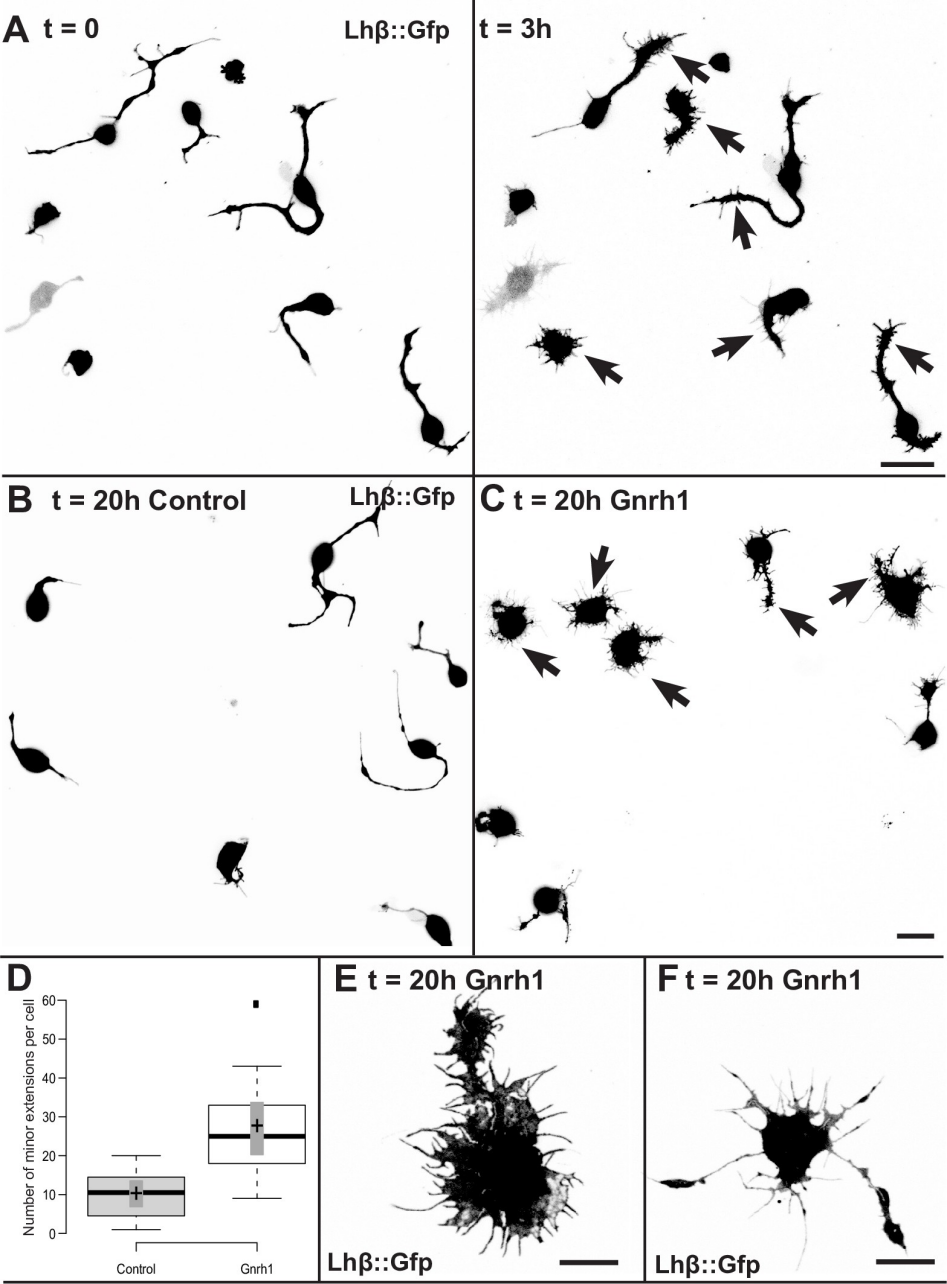
Lh cells in culture exposed to Gnrh1. (A) Planar confocal projections of Lh-cells 24 h after seeding, directly after (t = 0) and 3 h after addition of 10^−7^ M Gnrh1 (t = 3 h). See also S5 Video. (B) Planar confocal projection of non-treated Lh cells after 20 h additional incubation (t = 20 h control) and (C) Lh cells exposed to 10^−7^ M Gnrh1 for 20 h (t = 20 h Gnrh1). Note that this image is from another part of the dish than the image in A. (D) Boxplot showing number of minor extensions. Center lines show the medians; box limits indicate the 25th and 75th percentiles as determined by R software; whiskers extend 1.5 times the interquartile range from the 25th and 75th percentiles, outliers are represented by dots; crosses represent sample means; bars indicate 95% confidence intervals of the means. n = 16 control cells, n = 17 Gnrh exposed cells. Significant difference between means (*p<*0.001), calculated using t-test (two-tailed). (E and F) close-up images of two Lh cells after 20 h incubation in 10^−7^ M Gnrh1. Note the lamellar structures with a number of minor extensions (arrows). Scale bars represent 10 μm.

To assess whether Gnrh may affect the development of extensions, we followed Lh cells by time-lapse recordings for up to 20 h after adding Gnrh1 to the dish (Fig 8). The cells were seeded 24 h before the Gnrh1 exposure, and had thus already established extensions. Prior to Gnrh1 addition, the number and point of origin of the extensions were relatively stable, but the minor extensions were dynamic; growing and retracting (S5 Video). Almost immediately after Gnrh1 was added, the number of minor extensions started to increase, and after 3 h, the overall appearance of the Lh cells were already visibly different, with minor extensions sprouting from all parts of the cell (Fig 8A, time = 3 h). Exposure to Gnrh1 for 20 h resulted in 85 ± 3% of the cells (n = 120) developing a lamella on both cell soma and/or the major extension as shown in Fig 8C (arrows), and further in close-up images of two cells (Fig 8E and 8F). This is in contrast to the control cells that after 20 h did not change significantly in appearance (Fig 8B). In addition, Gnrh1 exposure resulted in a significantly higher number of minor extensions (27 ± 13 minor extensions per cell, n = 17) compared to control cells (10 ± 6 minor extensions per cell, n = 16, *p*<0.001). The number and length of major extensions varied immensely, but did not seem to change after 20 h of Gnrh exposure. On the other hand, the measurements were hampered by the lamellar structure of the soma and extensions, making the numbers too uncertain to analyze further.

## 4. Discussion

The established view regarding the morphology of endocrine cells from the anterior pituitary has been that they have a uniform, rounded shape with few or no extensions of any type. This view has been repeatedly challenged in recent years, as more refined imaging methods have become available [2, 4, 37, 38]. Our transgenic medaka line, in which the Lh cells are labeled with Gfp, display a surprisingly complex pattern of extensions, apparently connecting cells with each other. Although these extensions could be suspected to be an artifact associated with the primary culture protocol, our results show that similar structures are also clearly visible in intact pituitary and pituitary tissue slices. Also Lh cells from wild type medaka display similar extensions, including varicosity-like structures, indicating that these structures are not an artifact caused by the transgenic manipulation or a stress response to the Gfp. Extensions were also observed on some of the non-Lh cells, but these were not examined in this work. Our group has recently also developed a transgenic medaka line where the Fsh-producing cells are expressing a red fluorophore [17]. Some of these gonadotropes also display extensions, indicating that many, if not all, of the observed non-Lh cells in the present study are Fsh-cells. Nonetheless, the large majority of cells with extensions are Lh-cells, and for the few non-Lh cells with extensions, these are fewer and shorter.

Cellular extensions in non-neuronal cells have been subject of considerable attention lately [18, 25, 39–41]. However, as early as 1985, it was noted that mammalian gonadotropes could be stimulated to form extensions by GnRH [23]. The presence of gonadotrope extensions in mammals has later been confirmed [24, 25], but the extensions appear to be structurally quite different from the extensions on medaka Lh cells, for example, the reported extensions are shorter, and without swellings or branch-points [17, 20, 42]. To the best of our knowledge, structures similar to the extensions found on the unstimulated medaka Lh cells have not been reported elsewhere. Here we report two different types of extensions based on their appearance, defined as major and minor extensions.

**Fig 9:**
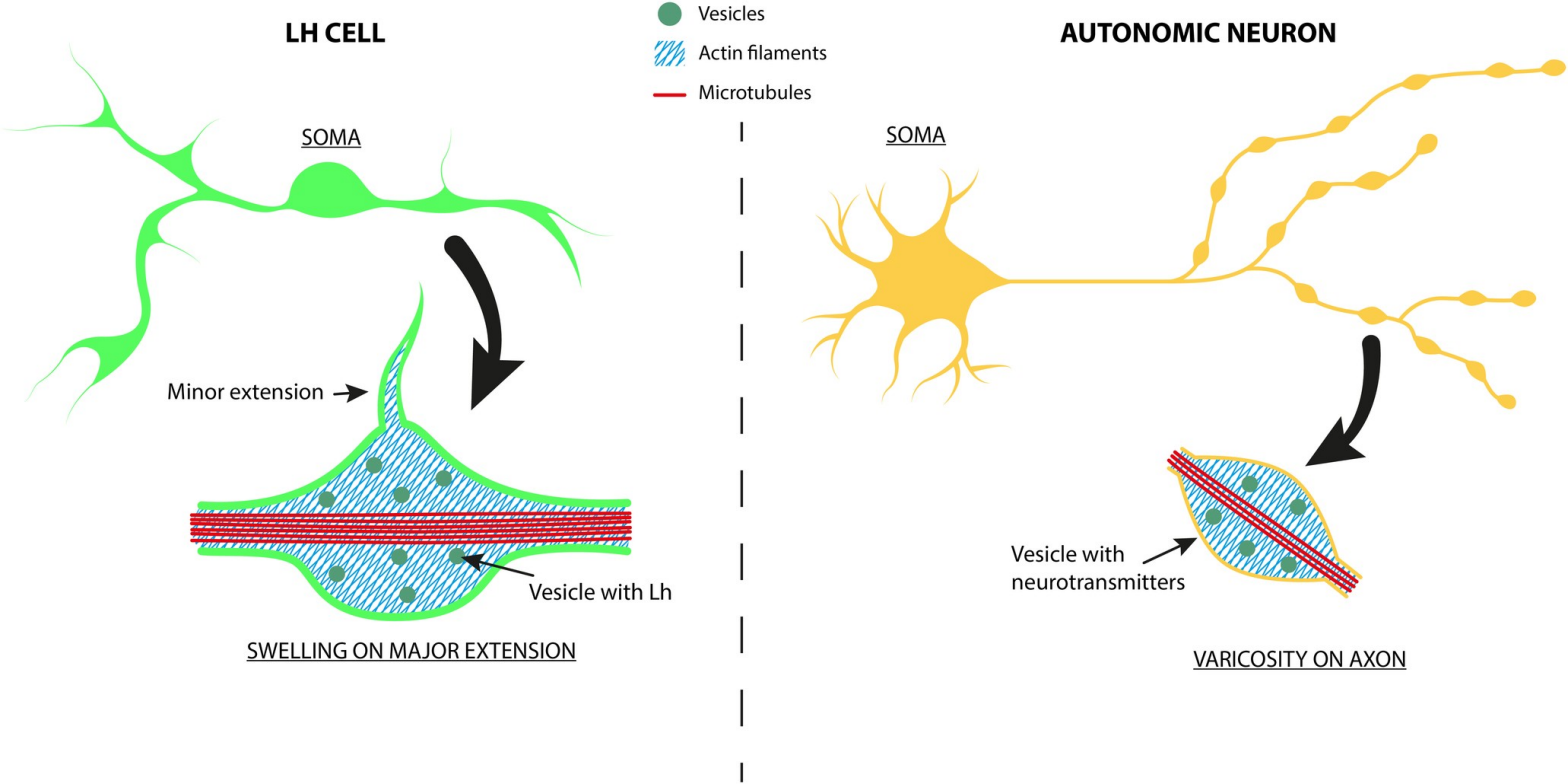
Scheme of an Lh cell from medaka and a typical post-ganglionic autonomous neuron, comparing the microanatomy of extensions vs neurites, and swellings vs varicosities. The anatomy of the Lh cell swelling must be considered a working hypothesis only, but is supported by the results in this paper.

The major extensions appear remarkably similar to neurites (Fig 9), regarding diameter, length and dependency on microtubules [43, 44]. In addition, both major extensions and neurites divide at distinct branching points, and display regularly spaced swellings containing large amounts of the main signaling substance [43, 45]. The transfer of Ca^2+^ signal, which plays an important role in regulating hormone release [46], from the soma via each swelling along the way to the terminal of an extension, together with the structural similarity of the swellings to axonal varicosities and the significant amount of hormone present in the swellings, opens up for the possibility that the extensions may have a role in secretion. In addition, the observations that the Ca^2+^ signal is neither transferred from extension to connected cell, nor propagated backwards to the cell soma, further indicate that the primary function of the extensions in vivo may be to facilitate hormone secretion from the swellings to nearby blood vessels. In mammals, it has been shown that distinct parts of neuronal axons and dendritic spines can contain components of the translational machinery, like ribosomes, indicating that proteins and peptides may be locally produced [47]. Furthermore, Gnrh has been shown to activate translational activity in a mouse gonadotrope cell line [48]. It would be very interesting to investigate whether the varicosity-like structures in medaka Lh cells may similarly contain ribosomes and locally produce and release Lh from these structures in response to Gnrh. In both mammals and teleosts there are indications that gonadotrope extensions may reach out to nearby blood vessels, resulting in more efficient release of hormone into the circulation [4, 16, 23–25, 49]. Our results indicate that this may also be the case in medaka, as some Lh cell extensions project into the perivascular space, offering the possibility of close contact between Lh cells and the microcirculation. However, whether these swellings really release Lh or other signaling molecules remain to be investigated. Likewise, such outreach to blood vessels would at the same time facilitate the response to Gnrh and other blood-borne signals. Whether the extensions display the necessary receptors for this putative function remains to be investigated. In intact pituitary we also see a lot of extensions not aiming at blood vessels. This lead us to speculate that these extensions could take part in a more diffusely organized functional network coordinating hormone production and release, but our results indicate that this is not the case, as we could not see any transfer of Ca^2+^ signals between cells connected via extensions in cell culture.

The minor extensions of the Lh cells are dependent on actin and are thus similar to the filopodia that originate from the growth cone of neurites [50, 51]. The lack of distinct swellings on the nocodazole- and the cytochalasin B-treated cells suggests that both microtubules and actin filaments are involved in the structure of the swellings, as has been reported for axonal varicosities [45, 52]. Intermediate filaments have been indicated to play a role in cytoskeletal rearrangements [53], and a possible interplay between microfilaments, microtubules, and intermediate filaments in Lh cell extensions would be worthy of further investigation. It would also be interesting, in the future, to assess whether the development of major extensions follows in the track of minor ones.

The most striking differences between the extensions observed on mammalian gonadotropes and medaka Lh cells are that in the latter, the extensions are longer, branched, have varicosity-like swellings, and the cytoskeleton is not only actin-based. It is important to note, however, that because cellular extensions are extremely fragile, some of the observed structural differences may reflect artifacts associated with differences in experimental protocols.

We clearly observed that Lh cells made physical contact with surrounding Lh cells through their extensions during the initial hours after seeding. This often resulted in a bridge formed between the two Lh cells, apparently strong enough to participate in drawing the two connected cells closer to each other, thus allowing them to cluster. The ability of filopodia to pull and push have attracted considerable attention [54], and future research may reveal the cytoskeleton dynamics behind the bridge shortening. In contrast to mammalian gonadotropes that are scattered throughout large parts of the anterior pituitary, teleost gonadotropes are clustered [8, 10, 14]. This means that there must be a targeted clustering of gonadotropes during development, even separating between Lh-expressing and Fsh-expressing cells. Lately, it has been demonstrated in medaka that active clustering may also occur during puberty, when a substantial proliferation of gonadotropes takes place, as prepubertal pituitaries show less clustering than adult pituitaries [20]. We could also observe potential contacts between Lh cells and non-Lh cells. The Lh cell extension often retracted after contact was made or orbited the non-Lh cell soma. We rarely observed formation of bridges or shortening of extensions after contact. Although the results show a tendency towards homotypic clustering using extensions, the process is occasionally also heterotypic. Since the non-Lh cells could not be further identified regarding cell type, we chose not to analyze these heterotypical contacts in detail. After three days in culture, the Lh cells dominated, amounting to almost 50% of the total. This is in contrast to the 15–17% reported from intact pituitary [28]. This discrepancy may be due to the dissociation process. As Lh cells are on the surface of the pituitary they will detach first and thus receive more gentle treatment, while cells further in will be detached after more mechanical work, potentially leading to more damage and thus more cell death. Thus, our analyses are focused on the Lh-Lh interactions, and the presented results on non-Lh cells may be biased.

Godoy and colleagues [55] have suggested that the morphological plasticity of gonadotropes has a developmental role in the organization of the anterior pituitary, as Gnrh induces cell migration in addition to formation of extensions. Indeed, there is evidence from mammalian studies that Gnrh induces plasticity of gonadotropes and their extensions by remodeling of the actin cytoskeleton and formation of lamellipodia [5, 25, 56]. Lamellipodia are dynamic domains involved in cell motility in several cell types [57, 58]. In a mice gonadotrope cell line, tonic GnRH has been shown to upregulate transcription of actin as well as other cytoskeleton components, making it possible not only to relocate cytoskeletal components during remodeling, but also increase the abundance of cytoskeletal structures [59]. We did not aim to extensively characterize such an effect on the Lh cells, but rather just confirm that also in medaka, the gonadotrope extensions are affected by Gnrh. A thorough analysis of the effect of Gnrh on extension formation, networking and clustering would be an interesting scope for future studies.

Previous research has shown that gonadotropes may be functionally linked in larger functional networks by gap junctions in both mammals and fish, resulting in efficient spread of signals regulating the hormone release [4, 60, 61]. Moreover, blocking this network in tilapia leads to lower Lh release [4]. Here, we observed an extensive interconnectivity of Lh cells via the extensions, but Ca^2+^ signals were not transferred from one cell to another through the extensions, and further studies are necessary in order to determine whether the Lh cells communicate via extensions in other ways, or whether the contacts are merely structural, closed-ended. However, we observed an effective transfer of Ca^2+^ signals between soma-to-soma connected cells that may be explained by the presence of gap junctions in the soma-soma connections, but not in the extensions. While the presence and the role of gap junction remains to be investigated in medaka, this study strongly suggests that Lh cells need to be soma-soma connected to allow spread of regulating signals, and thus that the clustering process taking place through the extensions has a primordial role in the establishment of this important communication network. Importantly, we recently demonstrated that not only do Lh cell transfer the Ca^2+^ signal to other contacting Lh cells, but we observed that Lh cells readily transfer the Ca^2+^ signal to Fsh cells as well [17]. Whether Lh cells also transfer signals to other cell types than gonadotropes, is an ongoing study in our group. Although Lh cells can communicate with Fsh cells and potentially also other pituitary cell types, the clustering process and formation of functional tight networks does not appear randomly, since both physical contacts and transfers of Ca^2+^ signal are dominated by Lh-Lh cell combinations in our cell culture studies.

Finally, one may speculate that the extensions have several functions. During development and reproductive remodeling of the pituitary, extensions may be particularly important for facilitating clustering and formation of tight functional networks with soma-soma contact points. Some of the extensions that remain in the adult pituitary may also contribute to coordinating the Lh population by some hitherto unknown mechanism, while other extensions may keep the cells in close contact with the microcirculation. While speculating, we must keep in mind that conditions found in cell cultures often do not transfer to the conditions *in vivo*. We demonstrate that Lh cells *in vivo* have long extensions with Lh-containing swellings, but cannot conclude on their *in vivo* functions based on the current results from cell culture. More advanced experiments on intact pituitary are necessary. Future studies will hopefully reveal the functional importance of both minor and major extensions and their swellings.

## Supporting information

S1 Fig Determining the 405 nm laser uncaging specificity, related to Fig 8. (A and B) Pseudo colored images of relative levels of cytosolic Ca^2+^ where blue represents basal levels and red peak Ca^2+^ levels. (A) The laser was targeted to about 5 micrometers outside the cell soma. (B) The laser was targeted directly on the cell soma of one cell in a cluster. (C) Corresponding Ca^2+^ traces from A and B with changes in fluorescence (ΔF) divided by the average intensity of the first 15 frames (F) as a function of time. Purple vertical bar represents the time point of uncaging. Blue trace represents the negative control in A and black trace the positive uncaging in B. The numbers represent each of the pictures in A and B. (D) Images of the laser spot in A and B. Pseudo coloring of the scattered light with white representing the saturation point were the laser is able to activate and uncage NP-EGTA.
(TIF)

S2 Fig Lhb immunostaining of live pituitary tissue from wild type medaka. Confocal fluorescence images (5 micrometers z-stack projections) from pituitary sections in WT medaka, labeled for LHb by immunofluorescence, showing Lh cell extensions (arrows) and the blebs along the extensions (arrow heads). Scale bars: 10 micrometers.
(TIF)

S3 Fig Flowchart of number of contacts initiated by an extension during the first 15 h after seeding. The criterium for analyzing a possible contact was that the distance between cells in the pair was more than 15 μm at time 0. The cells could be single or in a cluster. Total number of cells at time 0 was 171, where 52 cells were Lh cells. At 15 h, there were a total of 149 cells left, of which 51 were Lh cells.
(TIF)

S4 Fig Distribution of cells as single or in cluster over time (0–15 h). The green bars represent Lh cells, while the grey bars represent non-Lh cells. Different colour shades indicate the time after seeding, from dark to light; 0 h, 5 h, 10 h, 15 h. Total number of cells = 171, 158, 156, 149. Number of homotypic Lh clusters = 2, 6, 6, 7. Number of heterotypic clusters = 15, 14, 15, 14. Number of clusters with only non-Lh cells = 19, 12, 11, 10. It has to be noted that although the total number of heterotypic clusters is stable, the clustering process is dynamic, and cells are moving within and between clusters during this time period.
(TIF)

S1 Table Characteristics of extensions of Lh-cells treated with cytoskeleton inhibitors. (DOCX)

S1 Video Related to Fig 5B. Time-lapse video of primary culture during the first 17 hours after seeding. Frames are taken every 3 minutes. For scale bar and other details, see Fig 5.
(MP4)

S2 Video Time-lapse video of primary culture during the first 17 hours after seeding. Frames are taken every 3 minutes. Planar confocal projections of Gfp-labeled Lh cells were merged with transmitted-light images showing both Lh cells and non-Lh cells.
(AVI)

S3 Video Related to Fig 7A. Imaging of Ca^2+^ uncaging experiment showing propagation of Ca^2+^ signal through extension.
(MP4)

S4 Video Related to Fig 7B. Imaging of Ca^2+^ uncaging experiment showing propagation of Ca^2+^ signal across soma-soma border.
(MP4)

S5 Video Related to Fig 8A. Time-lapse video of the primary culture shown in Fig 9A for 10 minutes immediately before Gnrh1 exposure. Frames are taken every 30 s. For scale bar and other details, see Fig 8.
(AVI)

## Abbreviations

DiI: lipophilic carbocyanine dye
FSH/Fsh: Follicle-stimulating hormone protein (mammals/fish)
GFP: green fluorescent protein
GnRH/Gnrh: Gonadotropin releasing hormone protein (mammals/fish)
IQR: interquartile range
LH/Lh: Luteinizing hormone protein (mammals/fish)
Lhβ/Lhβ: Luteinizing hormone β subunit protein (mammals/fish)
Lhb/lhb: Luteinizing hormone β subunit gene (mammals/fish)
