# Supplementary figures and images for: Long extensions with varicosity-like structures in gonadotrope Lh cells facilitate clustering in medaka pituitary culture
Source: PLoS One. 2021 Jan 28;16(1):e0245462. doi: 10.1371/journal.pone.0245462 (PMC7842944; doi:10.1371/journal.pone.0245462)

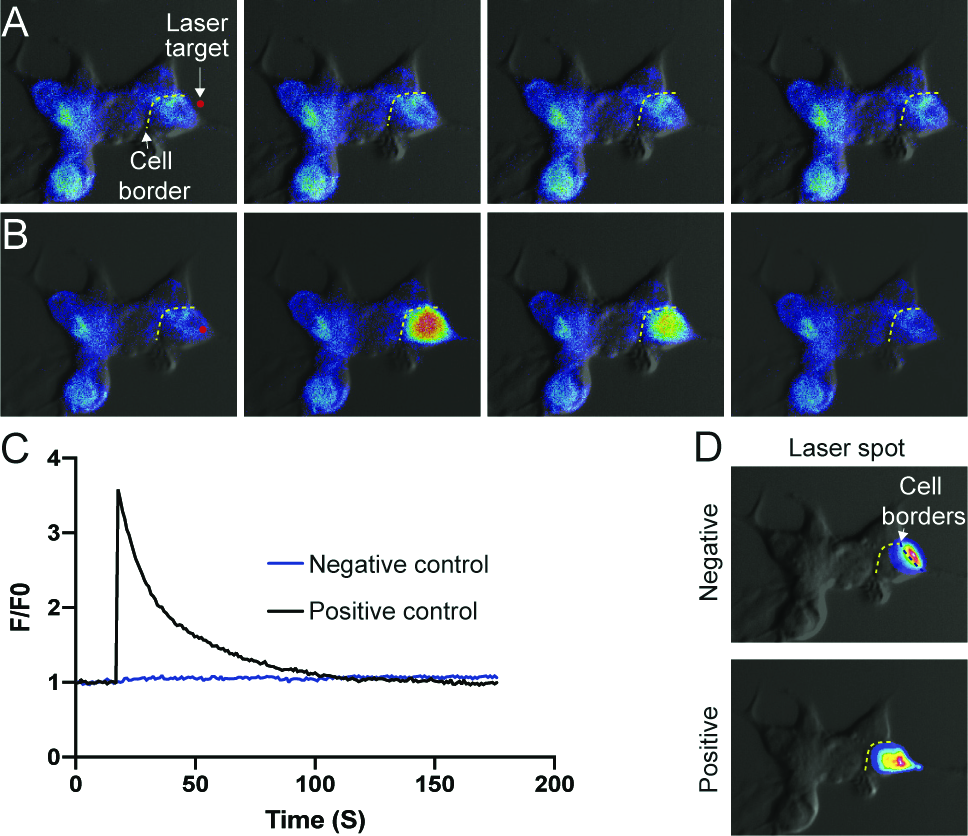

Supplement: S1 Fig — (A and B) Pseudo colored images of relative levels of cytosolic Ca2+ where blue represents basal levels and red peak Ca2+ levels. (A) The laser was targeted to about 5 micrometers outside the cell soma. (B) The laser was targeted directly on the cell soma of one cell in a cluster. (C) Corresponding Ca2+ traces from A and B with changes in fluorescence (ΔF) divided by the average intensity of the first 15 frames (F) as a function of time. Purple vertical bar represents the time point of uncaging. Blue trace represents the negative control in A and black trace the positive uncaging in B. The numbers represent each of the pictures in A and B. (D) Images of the laser spot in A and B. Pseudo coloring of the scattered light with white representing the saturation point were the laser is able to activate and uncage NP-EGTA. (TIF) [file pone.0245462.s001.tif]

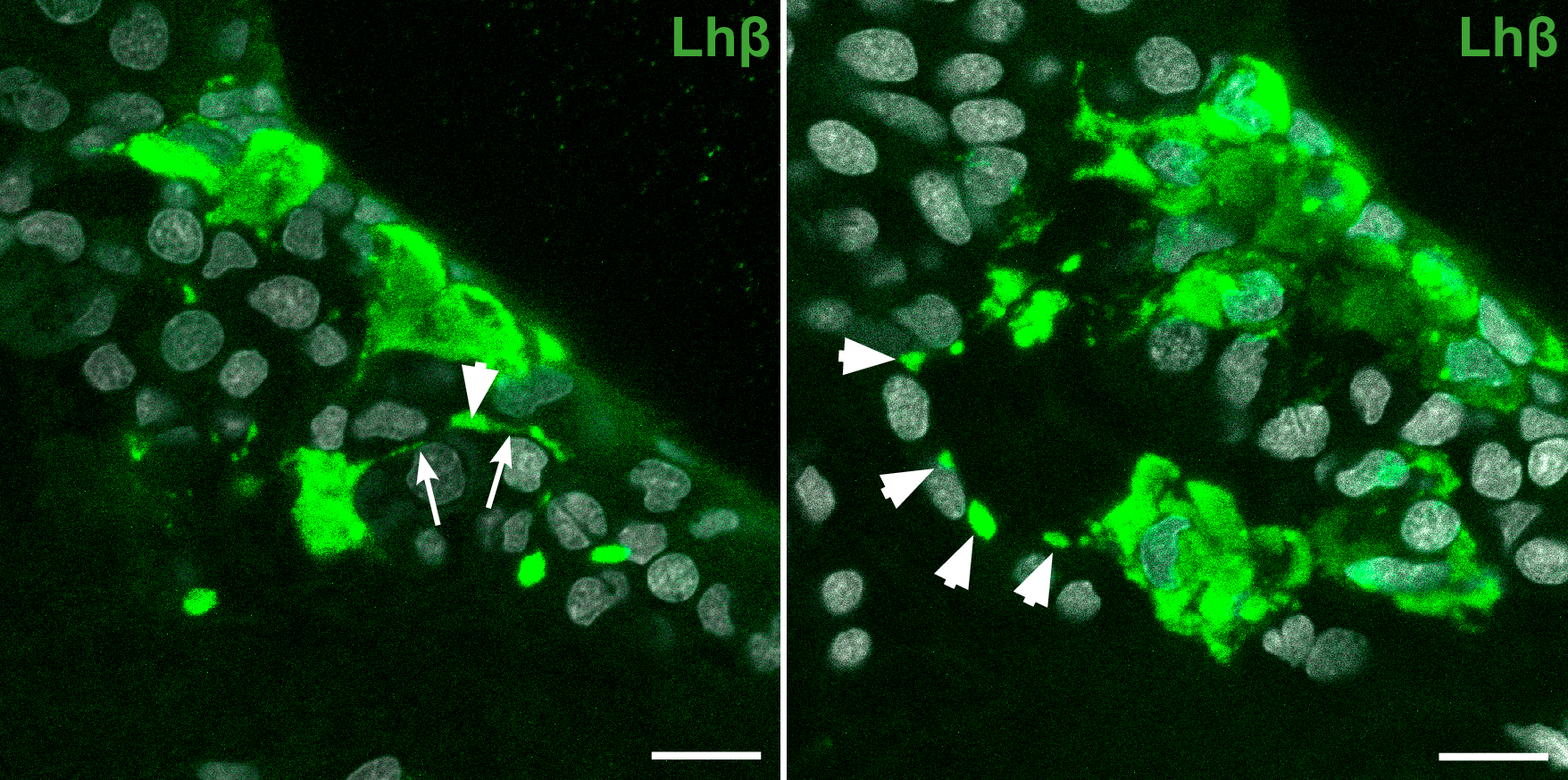

Supplement: S2 Fig — Confocal fluorescence images (5 micrometers z-stack projections) from pituitary sections in WT medaka, labeled for LHb by immunofluorescence, showing Lh cell extensions (arrows) and the blebs along the extensions (arrow heads). Scale bars: 10 micrometers. (TIF) [file pone.0245462.s002.tif]

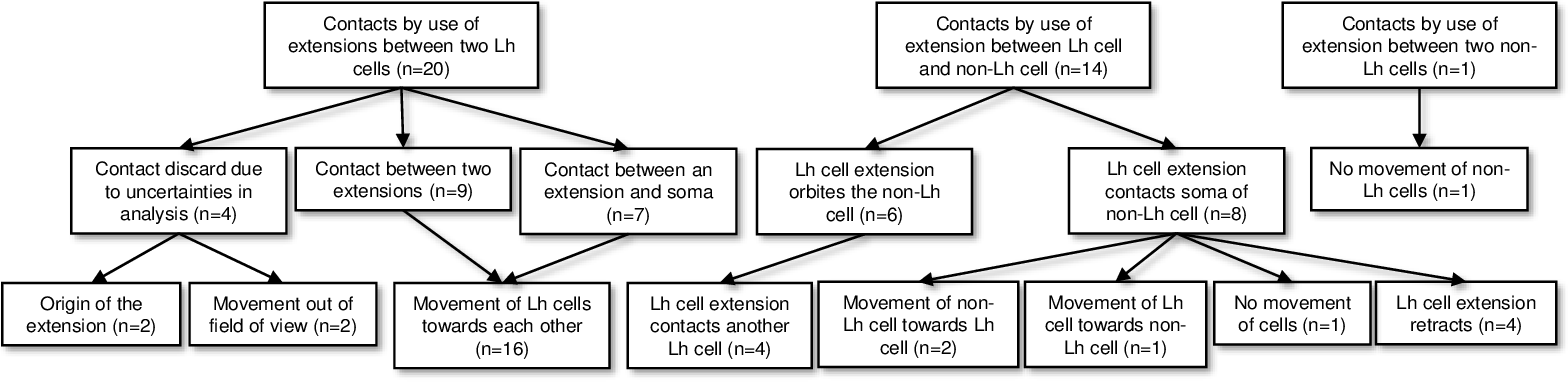

Supplement: S3 Fig — The criterium for analyzing a possible contact was that the distance between cells in the pair was more than 15 μm at time 0. The cells could be single or in a cluster. Total number of cells at time 0 was 171, where 52 cells were Lh cells. At 15 h, there were a total of 149 cells left, of which 51 were Lh cells. (TIF) [file pone.0245462.s003.tif]

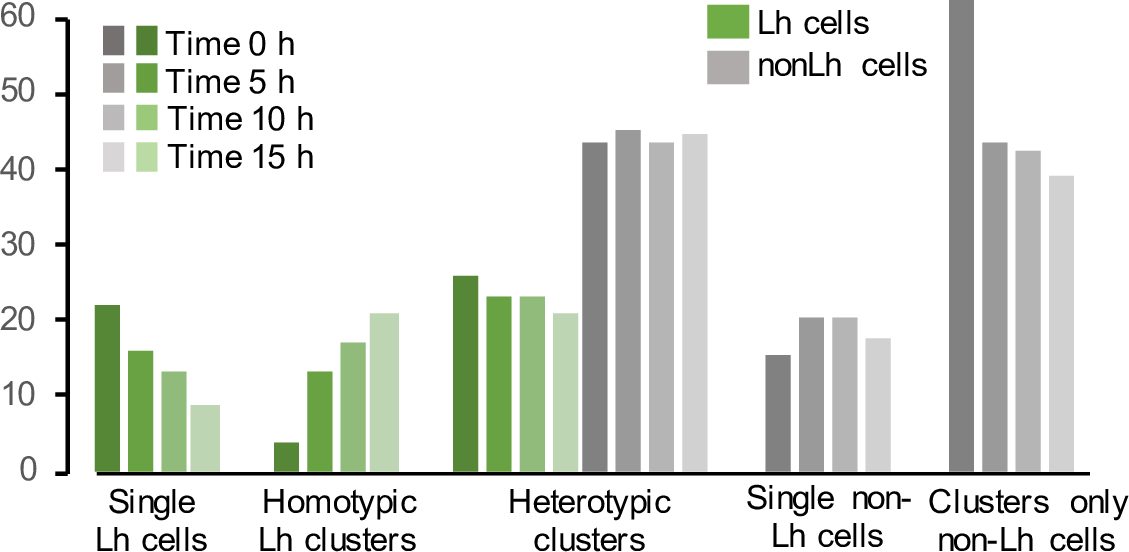

Supplement: S4 Fig — The green bars represent Lh cells, while the grey bars represent non-Lh cells. Different colour shades indicate the time after seeding, from dark to light; 0 h, 5 h, 10 h, 15 h. Total number of cells = 171, 158, 156, 149. Number of homotypic Lh clusters = 2, 6, 6, 7. Number of heterotypic clusters = 15, 14, 15, 14. Number of clusters with only non-Lh cells = 19, 12, 11, 10. It has to be noted that although the total number of heterotypic clusters is stable, the clustering process is dynamic, and cells are moving within and between clusters during this time period. (TIF) [file pone.0245462.s004.tif]
