# Supplementary material for: Long extensions with varicosity-like structures in gonadotrope Lh cells facilitate clustering in medaka pituitary culture
Source: PLoS One. 2021 Jan 28;16(1):e0245462. doi: 10.1371/journal.pone.0245462 (PMC7842944; doi:10.1371/journal.pone.0245462)
Supplement: S1 Table — (DOCX) [file pone.0245462.s005.docx]

**S1 Table. Characteristics of extensions of Lh-cells treated with cytoskeleton inhibitors.**

|  |  | Descriptive | | | |
| --- | --- | --- | --- | --- | --- |
|  |  | Diameter | Length | Swellings | Branching |
| Extensions in cytochalasin B treated cells | | 0.84 ± 0.22 | 8.32 ± 7.27 | No* | 21.6 ± 18.9 |
| Extensions in nocodazole treated cells | | 0.29 ± 0.10 | 5.69 ± 4.44 | No | 29.1 ± 9.4 |
| Major extensions in control cells | | 0.62 ± 0.16 | 13.05 ± 9.56 | 88.2 ± 10.4 | 70.1 ± 9.4 |
| Minor extensions in control cells | | 0.22 ± 0.05 | 4.24 ± 2.66 | No | No |
| The analyses are from cells 6 h after seeding. All values are from three independent cultures and presented as mean ± SD. Data are from 20 control cells with 66 and 69 major and minor extensions respectively, 27 cytochalasin B treated cells with 84 extensions, and 21 nocodazole treated cells with 125 extensions. *The extensions show some thicker parts, but no distinctive swellings. | | | | | |
